# Clinical and demographic profile of admitted victims in a tertiary hospital after the 2015 earthquake in Nepal

**DOI:** 10.1371/journal.pone.0220016

**Published:** 2019-07-18

**Authors:** Maria Moitinho de Almeida, Joris Adriaan Frank van Loenhout, Sunil Singh Thapa, K. C. Kumar, Benjamin-Samuel Schlüter, Ravikant Singh, Xavier Banse, Dan Putineanu, Deepak Prakash Mahara, Debarati Guha-Sapir

**Affiliations:** 1 Centre for Research on the Epidemiology of Disasters (CRED), Institute of Health and Society, Université catholique de Louvain, Brussels, Belgium; 2 Department of Orthopedics, Institute of Medicine, Tribhuvan University Teaching Hospital, Maharajgunj, Kathmandu, Nepal; 3 Tribhuvan University Teaching Hospital, Maharajgunj, Kathmandu, Nepal; 4 Doctors For You, Mumbai, Maharashtra, India; 5 Service d’orthopédie et de traumatologie de l’appareil locomoteur, Cliniques Universitaires Saint-Luc, Brussels, Belgium; Medical University Graz, AUSTRIA

## Abstract

**Background:**

In 2015, an earthquake killing 9,000 and injuring 22,000 people hit Nepal. The Tribhuvan University Teaching Hospital (TUTH), a reference tertiary hospital, was operational immediately after the earthquake. We studied the profile of earthquake victims admitted in TUTH and assessed what factors could influence hospital length of stay.

**Methods:**

An earthquake victim dataset was created based on patient records, with information on sex, age, date of admission and discharge, diagnosis, and surgical intervention. We performed an initial descriptive overview of the earthquake victims followed by a time-to-event analysis to compare length of hospital stay in different groups, using log rank test and cox regression to calculate Hazard Ratios.

**Results:**

There were in total 501 admitted victims, with the peak of admissions occurring on the fifth day after the earthquake. About 89% had injury as main diagnosis, mostly in lower limbs, and 66% of all injuries were fractures. Nearly 69% of all patients underwent surgery. The median length of hospital stay was 10 days. Lower limb and trunk injuries had longer hospital stays than injuries in the head and neck (HR = 0.68, p = 0.009, and HR = 0.62 p = 0.005, respectively). Plastic surgeries had longer hospital stays than orthopaedic surgeries (HR = 0.57 p = 0.006). Having a crush injury and undergoing an amputation also increased time to discharge (HR = 0.57, p = 0.013, and HR = 0.65 p = 0.045 respectively).

**Conclusions:**

Hospital stay was particularly long in this sample in comparison to other studies on earthquake victims, indirectly indicating the high burden TUTH had to bear to treat these patients. To strengthen resilience, tertiary hospitals should have preparedness plans to cope with a large influx of injured patients after a large-scale disaster, in particular for the initial days when there is limited external aid.

## Introduction

Earthquakes are large-scale, sudden-onset disasters, which cause widespread damage [1]. Between 2000 and 2018, there were 520 earthquake events with relevant human impact worldwide, with nearly two thirds having occurred in the Asian continent [2]. Health impacts of earthquakes depend not only on their magnitude and potential secondary effects, such as tsunamis or landslides [3], but also on characteristics of the community: built environment, time to rescue, availability of physicians and hospital beds, and prior training of lay uninjured survivors [3, 4].

Injuries are the most important cause of earthquake-related morbidity, consisting mostly of soft tissue and orthopaedic injuries, particularly fractures. These injuries are more frequent in the extremities, mostly lower limbs, and are also prone to wound infections [3]. Crush injuries are also a classic feature of earthquakes, and can develop into the potentially fatal crush syndrome [4–6]. Other causes of morbidity include respiratory diseases, usually related to inhalation of particulate matters [5], and cardiovascular diseases, such as hypertension and ischemic heart disease [7, 8]. Displacement-related outbreaks of communicable diseases and exacerbation of chronic conditions also contribute to earthquake morbidity [5]. Non-orthopaedic injuries to the head, chest, and abdomen have a lower likelihood of survival, and therefore they account for a minority of injuries treated in survivors [6]. Children and elderly, as traditional vulnerable groups as they are, are at higher risk of dying, as well as people with disabilities and comorbidities. Most deaths occur due to building collapse, and being indoors increases risk of death [4, 5].

On Saturday April 25th 2015, at 11:56 in the morning, Nepal was hit by an earthquake with a magnitude of 7.8 on the Richter scale, killing 9,000 people and injuring 22,000. The epicentre was in the Gorkha district, about 76 km northwest of the capital Kathmandu, but neighbouring districts were heavily affected [9]. Within hours, the Nepali Government made an official request for international assistance [10]. Several aftershocks occurred thereafter, with the strongest one taking place on May 12th. The Tribhuvan University Teaching Hospital (TUTH), in Kathmandu, is a reference hospital for the entire country. Established with a grant aid from Japan, it was built with Japanese earthquake resistance standards. In 2014, TUTH became part of the Hospital Preparedness for Emergencies (HOPE) project, having received staff training and designed a disaster management plan [11]. Immediately after the 2015 earthquake, victims and their relatives reached TUTH to seek healthcare. The hospital staff quickly provided medical attention to these victims, with immediate admissions and operation theatres running within two hours after the earthquake.

Understanding the profile of admitted earthquake victims is crucial to gain insight not only on health consequences of earthquakes, but also to understand what type of resources are mostly needed, in order to better prepare for such events. Literature on earthquake victims in low resource settings is mostly from foreign field teams who only arrive some days after the disaster, failing to picture the victims who reach the local health services in the first days after the earthquake [12, 13]. Since Nepal is a highly earthquake-prone country, understanding the immediate post-earthquake reality in local hospitals is extremely important to improve preparedness and response for the future. To our knowledge, the literature focusing on the Nepal earthquake victims treated within the country’s healthcare system is scarce [14]. The aim of our study was to describe the demographic and clinical profile of earthquake victims admitted to TUTH after the 2015 Nepal earthquake, and to understand which characteristics were associated with hospital length of stay.

## Materials and methods

### Data collection

Earthquake victims were defined as those patients admitted in TUTH with a clinical condition that could be directly related to the earthquake. These patients had records that identified them as such so the hospital could claim financial compensation from the Nepali government. The hospital staff later compiled these records into a database. In parallel, the government of Nepal created a standardized matrix to ensure comparability among different hospitals, and posteriorly filled it with TUTH patients’ information, but had a more conservative definition of earthquake victim, excluding cases such as acute stress disorder, deprivation syndromes, or acute exacerbation of chronic diseases. Some variables were reported in both datasets, but each dataset also contained unique information. In order not to lose this information, we linked both datasets based on six variables: Name, age, sex, living district, date of admission, and date of discharge. If there was a match in at least four out of six variables, we considered them the same subject. If there was a match in three of the six variables, we would verify the compatibility of diagnosis and treatment. If this was the same, we still considered the patient a match, otherwise not. Inconsistencies in variables between datasets (e.g. age, living district) were verified by hand search in patient records. If these records were not available, we used the information from the dataset generated by the hospital staff, as it collected data at an earlier stage. We included all victim admissions, including referrals from other hospitals, which occurred until October 2015. Readmissions and duplications were identified independently by two researchers, and those they agreed upon were removed from the victims dataset, as our study only intended to look at first admissions.

### Data description

Table 1 shows the variables in the original datasets that were used for this study. Age was categorized in four groups: 0–4; 5–14; 15–49; and 50 years or more. Delay of presentation was calculated as number of days between the first earthquake, on April 25^th^, and the admission. Length of hospital stay was computed by calculating number of days between admission and discharge. The main diagnosis for admission was entered as free text in the original files, and coded into the International Classification of Diseases, 10^th^ revision (ICD-10), by two independent researchers. If the coding did not coincide, they would discuss in order to reach a consensus. Only the first and main diagnosis was taken into account. Diagnoses were further converted into additional categorical variables, such as location of injury, presence of fracture, presence of crushing injury. Main body region of injury was classified in head and neck, lower limb, trunk (including chest, abdomen, and pelvis), and upper limb. If a person sustained several injuries in one same region, the injuries would be classified under that region. The medical specialty performing primary surgery was classified from an open text into a categorical variable. We created a binary variable for amputations and in-hospital deaths.

**Table 1:** Overview of variables in the original datasets.

Variable	Type	Possible values
Name*	Open text	
**Age**	Continuous	
**Sex**	Categorical	Male / Female
**Mechanism of injury**	Categorical	Object fall on patient
		Trapped in rubble
		Fall from height
		Other
**Date of Admission**	Date	
**Date of Discharge**	Date	
**Diagnosis**	Open text	
**Surgery Type**	Categorical	Neurosurgery
		Orthopaedics surgery
		Plastic surgery
		Wound care
		Other
**Surgery detail**	Free text	
**Comments on discharge**	Free text	

* this variable was solely used for merging datasets, and removed immediately after

### Data analysis

We first performed a descriptive analysis of selected variables, followed by a more thorough analysis of hospital length of stay. Statistical analysis of hospital length of stay is not straightforward. In general, the distribution curve is right-skewed and does not meet assumptions of Normality. In addition, in-hospital deaths make it uncertain when the discharge would have occurred. Time-to-event analysis, often known as survival analysis, allows inclusion of such cases [15]. As reported by Fenn and Davies and Sá et al. [16, 17], this is an appropriate method to study hospital length of stay as a dependent variable, particularly in small samples. We censored in-hospital deaths and used the non-parametric log-rank test to check for associations between length of hospital stay and the following variables: sex, age group, body region affected, surgery type, and the binary variables for fractures, amputations, and crush injuries. Based on these results, we calculated individual hazard ratios for each category of variables that had a significant log rank test. Finally, a multivariate Cox regression was computed, incorporating all covariates that showed significance in the previous steps. We checked for interaction between all variables, and performed regression diagnostics to the resulting model, using test of proportional hazards and a test of overall goodness of fit.

Data analyses were conducted using Stata 15.1 (StataCorp, College Station, Texas, USA). We considered a level of significance of p < 0.05.

### Ethics statement

For the purpose of this study, we only used secondary data from the hospital records. Identifiers were only used to merge the datasets, and were removed immediately after. In compliance with local requirements, we submitted this study protocol to the ethics committee of the Institute of Medicine of the Tribhuvan University. The requirement for an informed consent was waived, and we received clearance to undertake this study (Ref. 381(6-11-E)2/074/075).

## Results

### Descriptive overview

Our final victims dataset contained 501 individuals, with a mean age of 36.18 years (SD 21.58), ranging from 2 months to 87 years of age. Table 2 gives a descriptive overview of the included earthquake victims.

**Table 2:** Descriptive table of admitted earthquake victims.

	N(%)	Male, N(%)	Female, N(%)
**Total admissions**	501	242	254
**Age group**	495	242	253
0–4	18 (3.6)	10 (4.1)	8 (3.2)
5–14	67 (13.6)	30 (12.4)	37 (14.7)
15–49	266 (53.7)	139 (57.4)	127 (50.2)
> = 50	144 (29.1)	63 (26.3)	81 (32.0)
**Mechanism of injury**	170	85	85
Fall from height	30 (17.7)	14 (16.5)	16 (18.8)
Object fall on patient	90 (52.9)	44 (51.8)	46 (54.1)
Trapped in rubble	33 (19.4)	19 (22.4)	14 (16.5)
Slipped/tripped	15 (8.8)	7 (8.2)	8 (9.4)
Other	2 (1.2)	1 (1.2)	1 (1.2)
**Icd-10 groups**	494	242	252
Injuries	438 (88.7)	219 (90.5)	219 (86.9)
Contact with health services	16 (3.2)	9 (3.7)	7 (2.8)
Neurological	11 (2,2)	5 (2.1)	6 (2.4)
Respiratory	5 (1.0)	2 (0.8)	3 (1.2)
Circulatory	4 (0.8)	2 (0.8)	2 (0.8)
Mental disorders	4 (0.8)	2 (0.8)	2 (0.8)
Pregnancy-related	4 (0.8)	0	4 (1.6)
Other	12 (2.4)	3 (1.2)	9 (3.6)
**Location of injury**	399	195	204
Head and neck	73 (18.3)	39 (20.0)	34 (16.7)
Lower limb	195 (48.9)	107 (54.9)	88 (43.1)
Trunk	80 (20.1)	29 (14.9)	51 (25.0)
Upper limb	51 (12.8)	20 (10.2)	31 (15.2)
**Primary surgery**	345	170	174
Orthopaedics	226 (65.7)	117 (68.8)	109 (62.6)
Neurosurgery	40 (11.6)	16 (9.4)	24 (13.8)
Plastic surgery	31 (9.0)	13 (7.6)	18 (10.3)
Wound care	19 (5.5)	10 (5.9)	9 (5.2)
Other	28 (8.1)	14 (8.2)	14 (8.0)

Column percentages within variables are shown. ICD-10 group names were simplified for clarity. Sums do not always add up to total admissions due to missing items.

**Fig 1:**
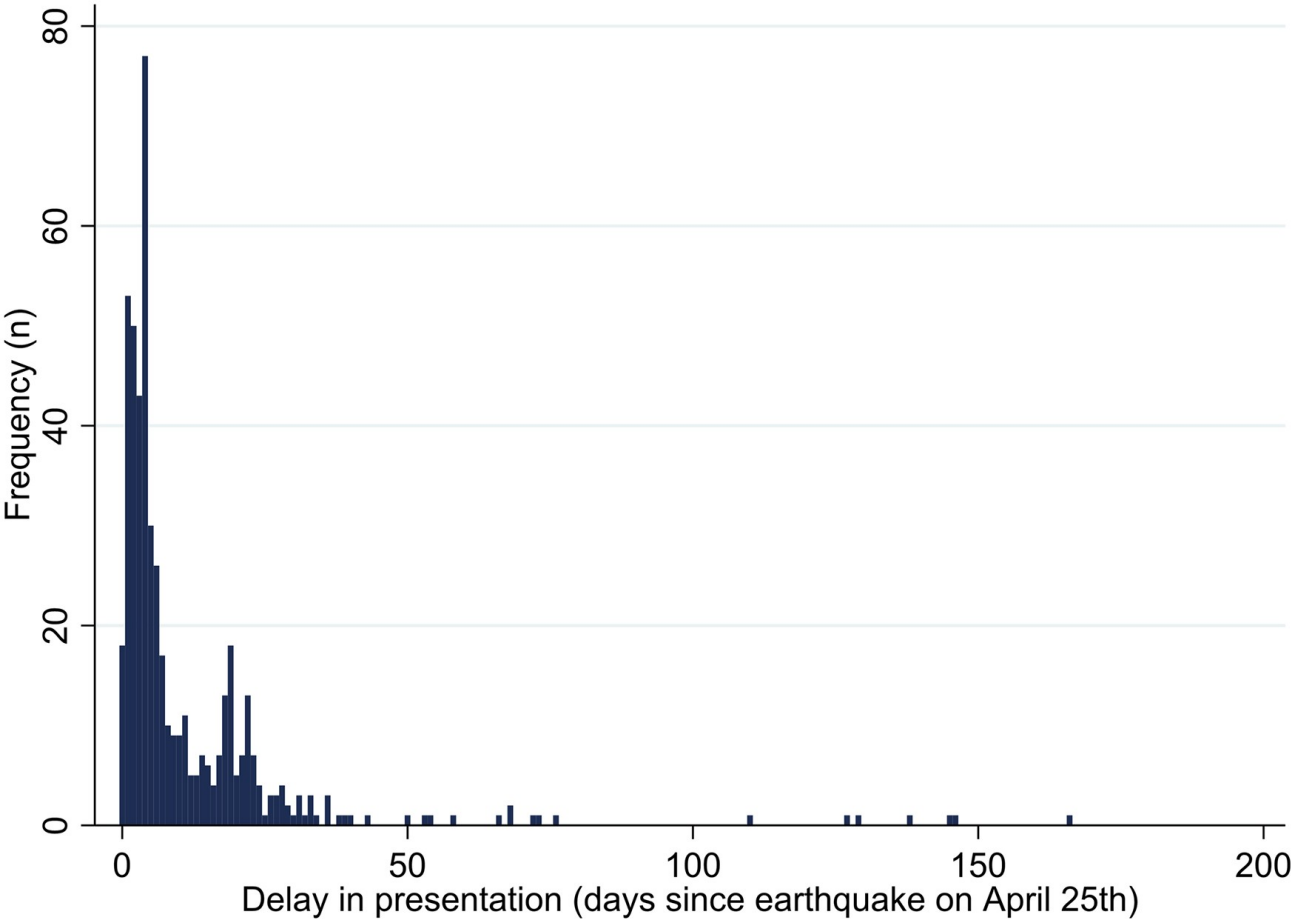
Distribution of delay in presentation. Delay in presentation was calculated as the number of days between the first earthquake on April 25^th^ and the date of admission.

Delay in presentation ranged between 0 and 166 days after the first earthquake on April 25^th^. The median was five days with an interquartile range of three to 15 days, and the peak occurred on the fifth day after the earthquake, with 77 admissions (Fig 1).

**Fig 2:**
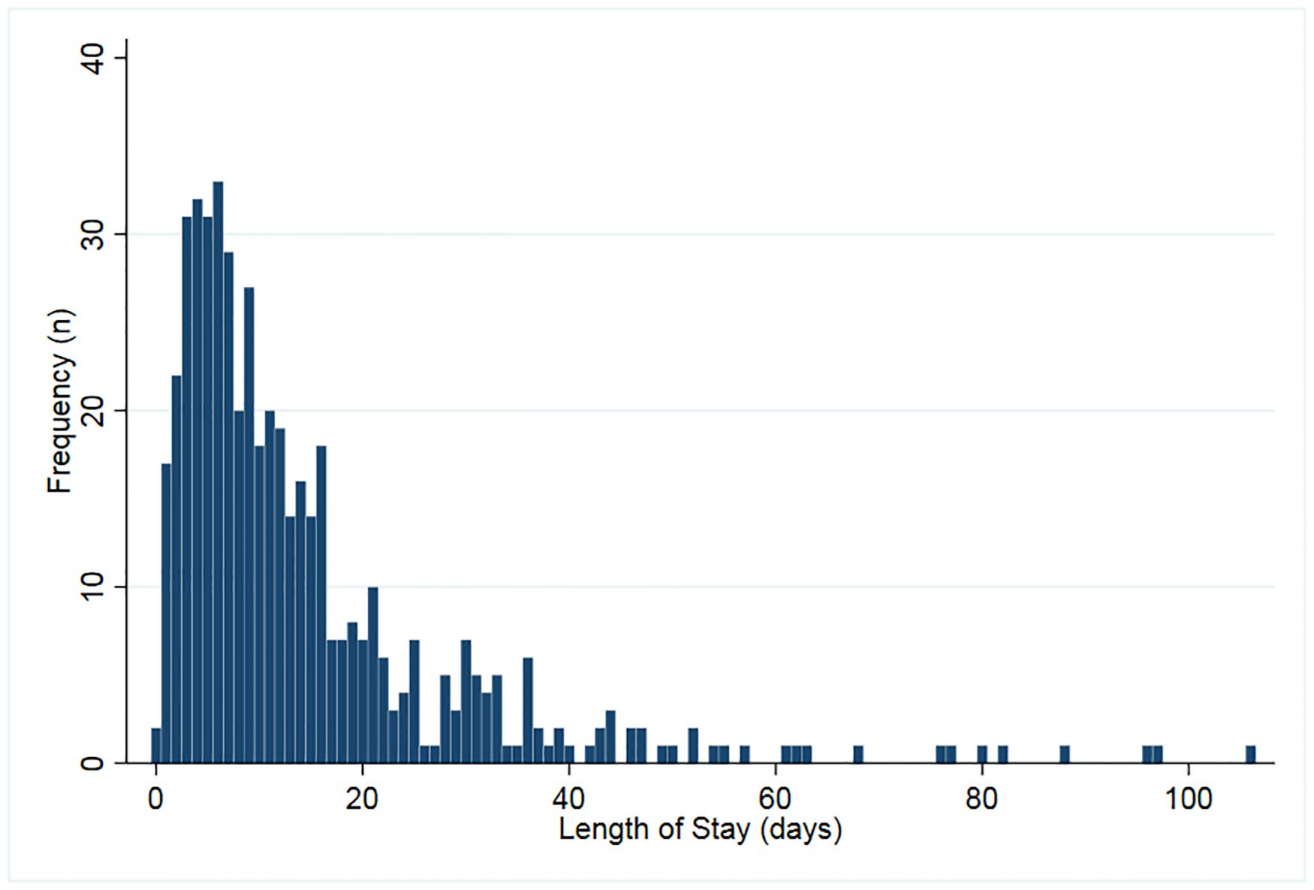
Distribution of length of hospital stay. Length of hospital stay corresponds to the number of days between the date of admission and date of discharge.

The mean length of hospital stay was 14.7 days (SD = 15.2), with a maximum of 106 days. The median length of stay was ten days, with an interquartile range of five to 18 days (Fig 2).

**Fig 3:**
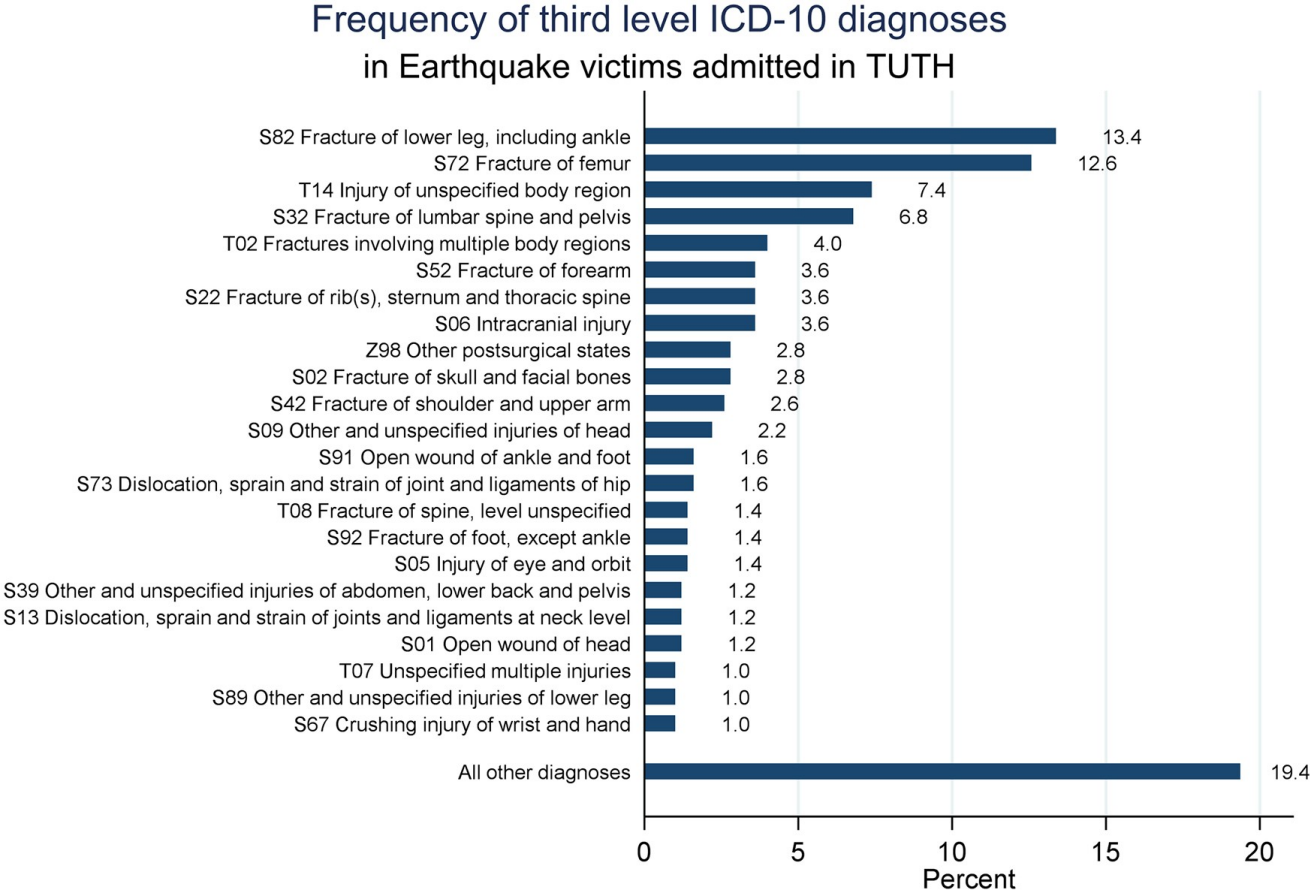
Frequency of primary diagnoses of admitted earthquake victims. Diagnoses with a relative frequency higher than 0.5% are shown, the remaining are aggregated under “All other diagnoses”.

Of all 501 admitted victims, 494 had diagnostic information (Table 2). The vast majority of the admitted victims (88.7%) had a diagnosis that fell in the “injury, poisoning and certain other consequences of external causes” group. Concerning third level ICD-10 diagnoses (letter and two digits), lower leg and femur fractures were the most common cause of admission, representing 26% of all diagnoses. These were followed by injuries to unspecified body regions and fractures of the lumbar spine or pelvis (Fig 3). More specifically, the most common diagnoses were fracture of the femur shaft (6.8% of all injuries), multiple lower leg fractures (5.5% of all injuries), and unspecified fractures of the lower leg (4.8% of all injuries). When looking at conditions that were not part of the injury group (n = 56), post-surgical states represent the majority of the cases (25% of all diagnoses not belonging to the injury group).

Fractures represented nearly two thirds of all injury cases (n = 288, 65.8%), with 49 compound fractures (17% of all fractures). Crushing injury was primary diagnosis for 21 admissions. About one third of all crushing injuries affected the upper limb (n = 6), one third affected the lower limb (n = 7), and one third occurred in an unspecified body region (n = 8).

About 69% of all admitted victims underwent at least one surgery (n = 345); this share was about 76% for fracture cases (n = 218). The majority of surgical interventions were orthopaedic, followed by neurosurgery, and plastic surgery (Table 2). About 61% of all primary interventions were considered major surgeries (n = 206), followed by intermediate surgeries (n = 119, 35%). Minor surgeries represented 14% of all surgeries in admitted victims (n = 15). Of all surgeries, there were 25 amputations (7.3%).

For 7% of the cases (n = 37), death was reported as an outcome, of which 20 were males and 17 were female. Most people who died were admitted one day after the earthquake (n = 10), and half were admitted within four days. Deaths occurred on average thirteen days after the admission, with a median of nine days of admission. Among in-patient deaths, 14 had a lower limb injury, seven sustained a head and neck injury, six a trunk injury, and four an upper limb injury. One person had a postsurgical state (craniotomy), and two were due to earthquake-related diseases of the nervous system.

### Time-to-event analysis

In the bivariate log rank tests, time to discharge was not associated with sex, age group, nor fracture. In contrast, it was significantly associated with main body region affected (p = 0.0026), presence of crush injury (p = 0.0093), having undergone an amputation (p = 0.0361), and surgery type (p = 0.0168).

Table 3 shows unadjusted individual hazard ratios (HR) for each category of variables that had a significant log rank test. Patients with injuries in lower limb and trunk had significantly lower hazards of discharge than head and neck, meaning they were hospitalized for a longer period. Plastic surgery was significantly associated with longer hospitalizations compared to orthopaedic surgery.

**Table 3:** Measures of association (unadjusted hazard ratios) of different characteristics with length of hospital stay.

Variable	*HR (95%CI)*	*Z*	*p*
**Body Region**			
Head and Neck	Ref		
Lower limb	0.68 (0.51–0.91)	-2.62	***0*.*009***
Trunk	0.62 (0.44–0.87)	-2.79	***0*.*005***
Upper Limb	0.99 (0.68–1.16)	-0.01	0.991
**Crushing**			
No	Ref		
Yes	0.57 (0.36–0.89)	-2.48	***0*.*013***
**Amputation**			
No	Ref		
Yes	0.65 (0.43–0.99)	-2.01	***0*.*045***
**Surgery Type**			
Orthopaedics	Ref		
Neurosurgery	0.90 (0.64–1.26)	-0.61	0.542
Plastic surgery	0.57 (0.38–0.85)	-2.76	***0*.*006***
Wound care	1.42 (0.87–2.34)	1.39	0.163
Other	0.84 (0.56–1.25)	-0.87	0.382

HR: hazard ratio; 95%CI: 95% confidence interval; Z: Z-score; p: p-value; Ref: reference category. Significant p-values (lower than 0.5) are presented in bold.

The four presented variables were incorporated in a multivariate model to calculate adjusted HR, which only included 291 observations due to missing values. The only significant finding was that injuries in the trunk region had lower hazards of discharge compared to head and neck (p = 0.005). However, this model did not meet the proportional hazards assumptions (chi-square = 28.47, 9 df, p = 0.0008), and there was significant evidence of a poor fit (p = 0.047). We checked for interaction terms between all variables, but these models were not significantly better.

## Discussion

The majority of the literature describing the profiles of treated victims from the Nepal earthquake is from foreign field teams, who usually arrive on site several days after the event [13, 18–20]. The experiences of such teams, albeit relevant, do not reflect the reality of the country’s health system and services, and fail to report on the difficult conditions in which local health actors must act in the first days after an earthquake [12]. Our study shows how a local health service dealt with earthquake victims immediately after the quake, with no time for preparations or resource planning to pursue life-saving activities. A first interesting finding is that the peak of victim admissions only occurred on the fifth day after the earthquake, an important feature for tertiary hospitals in seismic zones to take into account. Moreover, the date of admission of this sample had a wide range. One possible explanation is that some cases were referrals from other hospitals that were not able to provide the type of surgical care required. Secondly, in Nepal, access to surgical care was already challenged before the earthquake due to a poor road network and physical distance to appropriate health services. The earthquake worsened this situation by destroying paths up the mountain slopes, where the most severely affected villages were located, which could justify why many victims only reached TUTH after some days [21]. Furthermore, the regular aftershocks that followed the earthquake event, in particular the major one from May 12th, probably caused additional waves of injury. This may have also caused some of the later admissions.

More than half of victims with a reported mechanism of injury were hurt by an object falling on them. As described in the literature, building collapse is associated with higher mortality, and such victims are hence less frequently admitted, as they often die before reaching a health facility [4]. Despite incompleteness of mechanism of injury data, our results suggest that survivable injuries are often related to non-structural aspects of housing, such as falling or moving objects. Implementing best practices on non-structural aspects should be applied in people’s homes to avoid further injury, although this finding requires more in-depth research to be appropriately understood.

As expected, the vast majority of the earthquake victims were admitted primarily because of injuries, mostly orthopaedic, with fractures representing almost two thirds of all injuries. Lower limbs were the most common body location of injuries, and 17% of all fractures were compound. These findings are in line with previous studies on earthquake victims [6, 22, 23]. Fractures are conditions that allow patients to reach the hospital, and often justify hospital admission, since surgical intervention is usually warranted. Injuries that are non-orthopaedic, such as to the head, chest, and abdomen, are usually much more severe and deadly. Therefore, fewer individuals with such injuries reach health services and, as such, are not reflected in hospital admissions [6].

Children and adolescents under 15 years of age only accounted for 17.2% of the admissions, a share lower than the country’s age distribution in 2014, where they represented 33.4% of the population [24]. The earthquake occurred on a Saturday, a non-working day in Nepal, and during the day. Schools were closed and children were more likely to be outdoors, and hence, less severely affected than if the earthquake had occurred at night or on a school day. Furthermore, children in general are less prone to fractures than adults due to the properties of growing bone and, in the event of a fracture, the healing process does not require surgical intervention as often as in adults [25]. For this reason, fractures in children can be managed in the emergency department, resulting in less frequent hospital admissions. It is possible, however, that some children were sent to other treatment centres specifically set up to provide care to paediatric patients, although there is limited available information to support this. A study in a tertiary paediatric hospital in Kathmandu only reported on admissions nine days after the earthquake, without providing information on whether it was functioning before this date [26]. Nevertheless, given the disruption of the health system in Kathmandu, we believe that children in need of higher levels of care did not deviate significantly from TUTH.

The mean and median length of hospital stay of earthquake victims in TUTH, which were equal to 14.7 and ten days, respectively, are higher than in other studies. A study in a rural hospital in Nepal showed that earthquake victims had a median length of stay of eight days, as opposed to five days in non-earthquake patients. In another study comprising 1,878 admitted victims of the Wenchuan earthquake, their mean length of hospital stay was seven days, ranging from one to 120 days [14, 27]. Earthquake injuries are usually sustained by high levels of energy applied in the musculoskeletal system, which may increase injury severity and, as a consequence, delay discharge when compared to other conditions. Moreover, since TUTH is a reference hospital in the country, we expect more complicated cases to be sent there, which would explain why the mean and median length of hospital stays are higher than in other hospitals that were functional after earthquakes. Finally, it is also possible that some patients had their homes destroyed, or were from distant places with no available transport, and were kept longer in the hospital.

Our survival analysis indicates that trunk and lower limb injuries, crush injuries, undergoing plastic surgery, or undergoing an amputation, were associated with longer hospital stays. Injuries in the trunk region include pelvic and spine fractures, and such patients are generally more unstable and usually require definite treatment before being sent home. Lower limbs are relatively less vascularized than upper limbs [28], and need longer time to heal [29]. In addition, open fractures are more likely to occur in the leg, and such wounds have higher infection rates [30]. Besides, patients with lower limb injuries have more difficulties to walk, which possibly postponed some discharges in the context of collapsed houses and infrastructure in Nepal. On the other hand, crush injuries may develop into the potentially fatal crush syndrome, and are severe conditions requiring intense fluid therapy or even renal replacement therapy to ensure hydroelectrolytic balance [31]. Since it is an expectable condition in the aftermath of earthquakes, hospitals should be prepared to treat such patients, who will require a considerable amount of resources and longer hospital stays. Finally, wounds that require plastic surgery interventions are usually very prone to infection, particularly in earthquakes. Such infections need intravenous antibiotic therapy, forcing patients to stay in hospital. Also, reconstructive surgery often requires subsequent interventions, which naturally increases length of hospital stay [32].

Length of hospital stay is often described as the result of complex interactions between patients, accompanying physicians, and hospital management characteristics [16, 33]. Our findings suggest that earthquakes add to this complexity, as they trigger several injury admissions, cause serious disruptions in the surrounding environment such as in infrastructure or other health services, and disturb the normal functioning of the hospital. A previous study on admitted earthquake victims in China showed that length of hospital stay was not an appropriate proxy indicator for severe injury, but rather for major non-orthopaedic surgery and blood transfusion [27]. This points to the fact that length of hospital stay reflects hospital resource use after earthquakes.

### Limitations

This study had some limitations. Data were collected in chaotic conditions, primarily aimed at reporting to the government for financial compensation. We limited our analyses to the most reliable information available, which underwent several verifications. Completeness was lower for some specific variables, decreasing the power of statistical tests, and preventing us from including additional information, such as subsequent surgeries or co-morbidities, which could have enriched our analyses. Furthermore, we only analysed the main diagnosis as reported in the patient records. Although some conditions could be jointly classified as one ICD-10 code (e.g. multiple fractures in upper limb), some were left out from the analysis due to limitations in categorization, which was done by two independent researchers. As a consequence, it is likely that a relevant share of injuries were not taken into account.

We included earthquake victims who were admitted at TUTH at any stage, and our analysis contains referrals from other hospitals. However, because this information was not systematically collected, we cannot be entirely certain of which individuals were referrals. It is also not possible to affirm with certainty which victims were affected during the first earthquake or in its aftershocks. In addition, we lacked reliable information on posterior readmissions, which would have provided more insights on the long-term consequences for patients.

## Conclusions

Our study attempted to use existing data from a unique setting in the most efficient way possible, providing useful information for tertiary hospitals to improve preparedness and response in the event of an earthquake. There was a high influx of earthquake victims coming to TUTH for several days, with the peak occurring on the fifth day only. With an increased length of hospital stay for earthquake victims compared to other settings, and some conditions that specifically need higher levels of care requiring longer admissions, TUTH faced an extremely high burden after the 2015 earthquake. To strengthen resilience, tertiary hospitals in countries with seismic risks should have preparedness plans to cope with large numbers of injured patients after earthquakes, anticipating that they will need considerable resources. In particular, there should be a strong focus on the initial days following the disaster, since there is a sustained influx of patients, but limited external aid.

In order to improve our understanding of this disaster and of how it affected hospital functioning, we are conducting additional studies focusing on gender differences, changes in patterns of hospital admissions, and on hospital staff experiences.
